# Case report: Severe hepatopathy following rivaroxaban administration in a dog

**DOI:** 10.3389/fvets.2024.1364677

**Published:** 2024-04-04

**Authors:** Allison P. Mosichuk, Candace Wimbish, Kristen Poplawski, Adam Birkenheuer, Karyn Harrell, Kursten V. Pierce

**Affiliations:** Department of Clinical Sciences, College of Veterinary Medicine, North Carolina State University, Raleigh, NC, United States

**Keywords:** rivaroxaban, hepatopathy, canine, anticoagulant, factor Xa inhibitor

## Abstract

Rivaroxaban, a specific factor Xa inhibitor and commonly utilized anticoagulant, has been known to cause hepatotoxicity and liver failure in humans. Although rivaroxaban is frequently used in veterinary medicine, hepatotoxicity has not been previously reported in dogs. The current case report describes a dog that developed severe hepatopathy following rivaroxaban administration for a large right pulmonary artery thrombus. An estimated 6-year-old spayed female mixed-breed dog developed anorexia and lethargy 9 days after rivaroxaban administration began. Subsequent labwork revealed severe hepatocellular hepatopathy, and rivaroxaban was discontinued. Additional diagnostics did not reveal an underlying etiology, although hepatic cytology could be consistent with a toxic injury. The hepatopathy and clinical signs improved after rivaroxaban was discontinued. The time to onset, type of hepatopathy, and time to resolution were all similar to those reported for human cases. This case provides precedence to advocate for improved and closer monitoring of dogs receiving factor Xa inhibitors. In cases of suspected hepatotoxicity with no other identifiable cause, a risk–benefit analysis should be performed, and discontinuation of rivaroxaban administration or alternative anticoagulant medications should be considered.

## Introduction

Rivaroxaban is a human-specific factor Xa inhibitor utilized for the prevention and treatment of thrombosis (1, 2). Factor Xa inhibitors target the clotting cascade at both the intrinsic and extrinsic pathways, leading to the reduction of thrombin and ultimately clot formation (3). Rivaroxaban competitively inhibits free and clot-bound factor Xa as well as inhibiting prothrombinase activity, thus creating a potent oral anticoagulant (4). In humans, rivaroxaban and other factor Xa inhibitors have many approved indications, including stroke reduction in patients with atrial fibrillation, treatment of deep vein thrombosis and pulmonary embolism, and prophylaxis following surgery (5). Although rivaroxaban is an effective anticoagulant, significant adverse effects, including bleeding, abdominal discomfort, back pain, anorexia, fever, and liver failure have been reported in humans (3, 6–9).

Rivaroxaban is prescribed off-label for anticoagulation in both dogs and cats in veterinary medicine (1, 10). Patients with a high risk of thrombosis include dogs with immune-mediated hemolytic anemia (IMHA), protein-losing nephropathy, or heartworm disease and felines with cardiomyopathy; however, there are numerous other conditions where antithrombotic drugs are indicated (10–12). While there are many anticoagulants used in veterinary medicine, evidence-based recommendations regarding specific treatment protocols are lacking (13). At this time, the recommended dose of rivaroxaban is 1–2 mg/kg/day orally in dogs (13). Rivaroxaban appears to be safe and well tolerated in dogs, with only minor bleeding and vomiting reported as adverse effects (1, 14, 15). However, it should be noted that the evidence of overall safety and tolerance is still quite limited in veterinary patients as compared to humans due to the limited number and small sample sizes of these studies. Unlike humans, rivaroxaban hepatotoxicity has not yet been reported in dogs.

The current case report describes a dog who developed severe hepatopathy following rivaroxaban administration. No other inciting cause was identified for the hepatopathy, which resolved after discontinuation of the rivaroxaban.

## Case presentation

**Figure 1:**
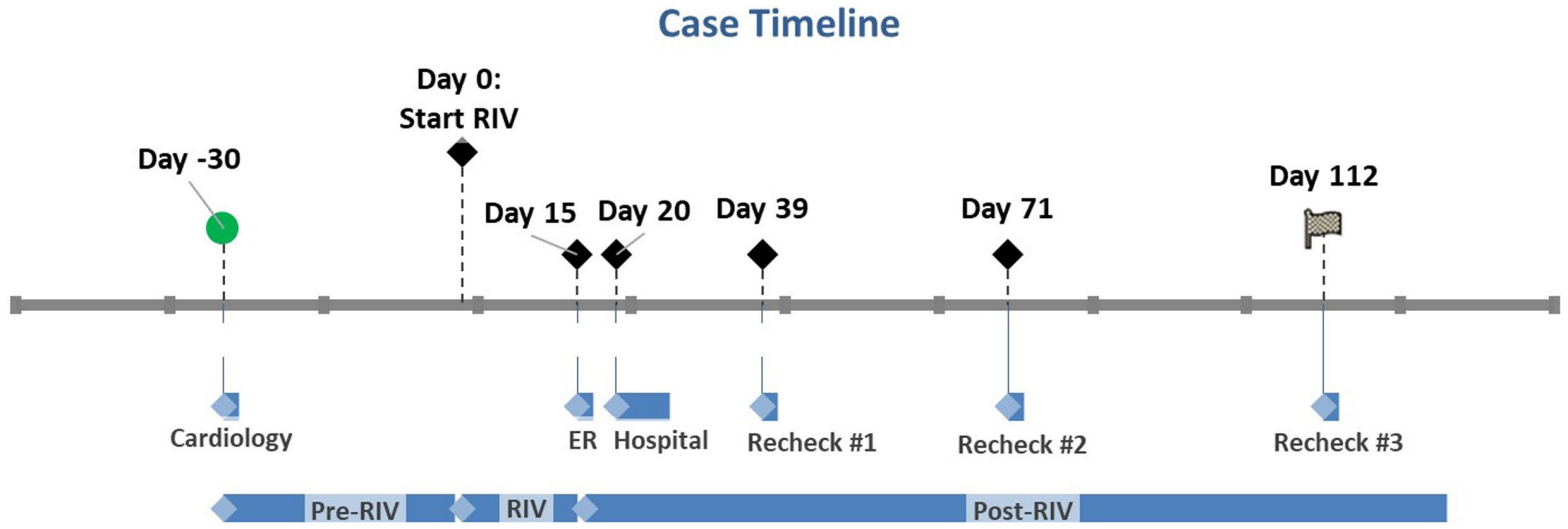
Case timeline where Day 0 marks the day rivaroxaban was started. Day - 30 marks the patient’s initial cardiology appointment. Day 15 marks the initial ER visit when rivaroxaban was discontinued. Days 20–27 mark the patient’s hospital stay. The subsequent recheck examinations are marked by Days 39, 71, and 112. RIV, Rivaroxaban.

An estimated 6-year-old spayed female mixed breed dog (weight: 21 kg) presented to the North Carolina State Veterinary Hospital Cardiology Service for evaluation of a new-onset heart murmur and generalized radiographic cardiomegaly (Day - 30; Figure 1). The heart murmur was first reported by the dog’s primary veterinarian at an annual wellness appointment 3 months prior. At the time of presentation, the dog was asymptomatic. There was a history of one episode of dyspnea characterized by significant expiratory effort and a normal respiratory rate (32 breaths per minute) approximately 2 months prior. This episode resolved within 2 h without treatment. The dog was adopted as a mature adult approximately 2 years prior with an unknown previous medical history. At the time of adoption, the dog tested positive for heartworm infection (*Dirofilaria immitis*) and exposure to *Borrelia burgdorferi* and *Ehrlichia* sp. (*Ehrlichia canis, Ehrlichia ewingii,* or *Ehrlichia chaffeensis*). The dog was previously treated at the time of adoption with an unknown heartworm treatment protocol and since tested negative. The dog was also treated with doxycycline (unknown dose and duration) for the *Borrelia* and *Ehrlichia* exposures.

**Figure 2:**
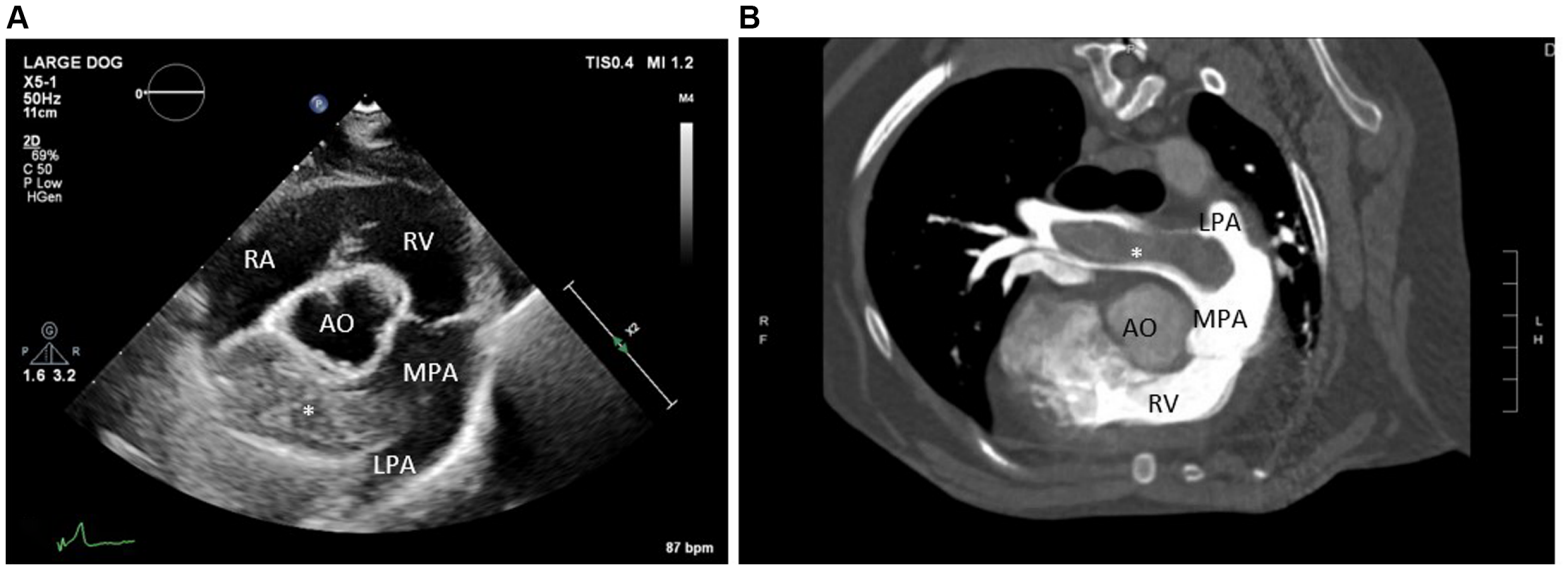
**(A)** Echocardiogram image (right parasternal short axis view at the level of the pulmonary artery) and **(B)** computed tomography images (transverse view) identifying a large thrombus within the right branch pulmonary artery. RA, Right atrium; RV, Right ventricle; MPA, Main pulmonary artery; AO, Aorta; LPA, Left branch pulmonary artery; ^*^large thrombus in the right branch pulmonary artery.

On physical examination, the dog was noted to have a grade II–III/VI right sternal systolic heart murmur. An echocardiogram was performed, which revealed moderate right ventricular enlargement, moderate tricuspid regurgitation (TR), and moderate pulmonary hypertension (TR pressure gradient ~60 mmHg). The most significant finding was an approximately 1.5 cm by 5.5 cm hyperechoic structure within the distal main pulmonary artery and right branch pulmonary artery (Figure 2A). These echocardiographic findings prompted additional workup including labwork (complete blood count, serum chemistry profile, urinalysis, urine protein to creatinine ratio, and IDEXX SNAP 4Dx Plus Test), kaolin-activated thromboelastography (TEG), and an anesthetized thoracic/abdominal computed tomography (CT) scan. Abnormal laboratory findings included a mildly elevated alanine transaminase (ALT 118 IU/L, RR 17–78 IU/L), mild proteinuria (urine dipstick), and a mild hypercholesterolemia (cholesterol 498 mg/dL, RR 151–348 mg/dL) (Table 1). The dog was noted to be persistently positive for antibodies against *Borrelia burgdorferi* and *Ehrlichia sp.* and was negative for heartworm antibodies. The TEG was within normal limits. On CT, an extensive, mineralized right pulmonary arterial thrombus and partially occlusive thrombi within the right middle, right caudal, and left caudal lobar arteries were noted (Figure 2B). The underlying prothrombotic condition was suspected to be secondary to her previously diagnosed heartworm disease. In light of her current pulmonary thromboembolism (PTE) and for the prevention of additional PTE, the dog was prescribed clopidogrel (1.8 mg/kg PO q24 h), with plans to start rivaroxaban (0.5 mg/kg PO q12 h).

**Table 1:** Serial serum biochemistry results, with Day 0 being when Rivaroxaban treatment was initiated.

Blood biochemistry results														
Test	Day−177	Day −30	Day 15	Day 20	Day 21	Day 22	Day 24	Day 25	Day 27	Day 39	Day 71	Day 112	RR	Units
Glu	152	89	91	114	95	94	119	142	120	102	96	117	75–126	mg/dL
BUN	23	17	13	5	3	3	2	1	3	15	18	16	11–27	mg/dL
Creatinine	0.6	0.5	0.4	0.7	0.3	0.4	0.3	0.3	0.3	0.6	0.6	0.9	0.5–1.4	mg/dL
ALT	175	118	2,688	2035	1,664	1790	1,090	907	633	760	126	134	17–78	IU/L
ALP	125	39	320	445	450	515	415	392	438	335	65	55	9–88	IU/L
AST	~	29	401	~	299	336	100	87	86	~	24	~	16–42	IU/L
GGT	1	5	49	~	44	73	60	53	55	37	3	2	0–4	IU/L
Total bilirubin	0.2	<0.2	0.2	0.7	0.5	0.4	0.5	0.5	0.3	0.3	<0.2	0.1	0.0–0.2	mg/dL
Cholesterol	226	493	613	~	403	406	275	241	252	230	438	178	151–348	mg/dL
Albumin	2.7	3.8	3.8	4.2	3.5	3.4	3.3	3.3	3.2	3	3.7	2.6	3.2–4.3	g/dL

Days –177 and –30 are a previous annual labwork panel and cardiology workup, respectively. Days 15 and 20–27 were during the initial emergency visit and internal medicine hospitalization, respectively. Days 39, 71, and 112 were post-hospitalization follow-up values. The normal reference ranges and units of measurement are provided. RR, Reference range; Glu, Glucose; BUN, Blood urea nitrogen; ALT, Alanine transaminase; ALP, Alkaline phosphatase; AST, Aspartate aminotransferase; and GGT, Gamma-glutamyltransferase.

Three days after starting clopidogrel (Day - 27), the dog developed severe, persistent diarrhea that was refractory to medical management with a bland diet and probiotics. The clopidogrel was discontinued after a total of seven doses, and the diarrhea significantly improved approximately 36 h later. After 7 days, the dog was trialed on a reduced dose (0.9 mg/kg PO q24 h) of clopidogrel; however, she again developed significant diarrhea 4 days later. At that time, clopidogrel was permanently discontinued (Day - 7). No laboratory data were obtained during this time. Rivaroxaban had not yet been started due to a delay in the owner receiving the medication. The dog was administered the first dose (0.5 mg/kg PO q12 h) of rivaroxaban 30 days after her initial presentation to the cardiology service and 7 days after her final dose of clopidogrel (Day 0; Figure 1).

At the scheduled recheck 5 days after starting rivaroxaban (Day 5), the dog was doing well at home with no recurrent respiratory signs, resolved diarrhea, and no noted adverse effects. The echocardiogram from this visit revealed similar findings compared to her previous visit (static size of the right pulmonary artery thrombus). Continued administration of rivaroxaban at the same dose was recommended, and the dog was scheduled for another recheck in 2 months.

The dog was re-presented through the North Carolina State Veterinary Hospital Small Animal Emergency Service 10 days after the cardiology recheck visit for progressive hyporexia and lethargy (Day 15; Figure 1). These signs had begun approximately 6 days prior (Day 9) after the dog had received 19 total doses of rivaroxaban. Her physical examination remained unchanged. Labwork (complete blood count and serum chemistry profile) was performed, which revealed a severe, primarily hepatocellular hepatopathy characterized by severe ALT elevation at 2,688 IU/L (RR 17–78 IU/L; Table 1). An abdominal ultrasound was performed by a board-certified radiologist, which was within normal limits with no reported abnormalities visualized. Outpatient care was elected by the owner. The dog was started on maropitant (2 mg/kg PO q24 h) and her rivaroxaban was discontinued due to concern for rivaroxaban-induced hepatotoxicity. Maropitant was administered for preemptive treatment and prevention of nausea, alleviation of visceral pain, and in hopes of improving food intake.

The dog remained anorexic at home and was re-presented through the North Carolina State Veterinary Hospital Small Animal Emergency Service 5 days after the previous emergency visit (Day 20; Figure 1). No additional clinical signs were reported. Repeat labwork showed a static hepatopathy (ALT 2035 IU/L; total bilirubin 0.7 mg/dL, Table 1). The dog was hospitalized and transferred to the North Carolina State Veterinary Hospital Internal Medicine Service the following morning. The dog was hospitalized for 7 days (Days 21–27). Repeat serum chemistry profiles were performed during hospitalization (Table 1).

Additional diagnostics were performed during the dog’s hospitalization including a coagulation profile, TEG, bile acids, ammonia, repeat hepatic ultrasound, liver/bile cytology, bile culture, and leptospirosis serology and PCR. The coagulation profile and TEG were consistent with mild thrombocytopenia with no evidence of hypercoagulability. Pre-prandial bile acids and ammonia were both within normal limits. Repeat abdominal ultrasound continued to show no significant changes in the liver, and aspirates of the hepatic parenchyma and bile from the gall bladder were performed. The bile sample was cytologically unremarkable. The liver cytology, performed by a board-certified clinical pathologist, showed hepatocellular atypia with glycogen vacuolar change, most consistent with toxin insult/injury, hepatic neoplasia, or regeneration. No growth was reported in the bile culture. Leptospirosis serology and PCR were not consistent with current or previous infection.

During the dog’s hospital stay, she was maintained on 50 mL/kg/day of 0.45% NaCl with KCl additive intravenously. The dog received ondansetron (0.5 mg/kg IV q8 h), maropitant (1 mg/kg IV q24 h), and capromorelin (3 mg/kg PO q24 h) for nausea, prevention of vomiting, the potential benefit of alleviating visceral pain, and appetite stimulation, respectively. The dog was started on N-acetylcysteine (140 mg/kg IV loading dose, then 70 mg/kg IV q6 h) and ursodiol (5 mg/kg PO q12 h) for liver support. After obtaining liver and bile aspirate results, enrofloxacin (10 mg/kg IV q24 h) and ampicillin/sulbactam (30 mg/kg IV q8 h) were also started on the fourth and fifth days of hospitalization, respectively (Days 24 and 25). Due to continued anorexia despite current anti-nausea therapy, the ondansetron dose was increased (1 mg/kg IV q8 h) and a metoclopramide CRI (1 mg/kg IV loading dose then 2 mg/kg/day IV) was initiated.

Over time, the liver values improved (Table 1); however, the dog continued to be anorexic. An NG tube was placed, and tube feedings were tolerated. The dog’s appetite slowly improved, and by discharge, she was eating small amounts. At the time of discharge, the seventh day of hospitalization, liver values had significantly improved (ALT 600; total bilirubin 0.3; Table 1). The dog was discharged with trazodone, gabapentin, ondansetron, denamarin, mirtazapine, and amoxicillin/clavulanate. The dog’s appetite began to improve at home; however, the owner was unable to administer any of the prescribed medications. Repeat labwork with her primary care veterinarian the week after discharge showed static to mildly improved values (Day 39; Figure 1; Table 1).

The dog returned to the North Carolina State Veterinary Hospital Cardiology Service on Day 71 for a recheck echocardiogram (Figure 1). Her appetite had significantly improved, and she was otherwise doing well. A serum chemistry profile showed significantly improved liver values (Table 1). Her echocardiogram was static from her previous imaging.

Labwork performed on Day 112 revealed that the liver values had returned to baseline (Figure 1; Table 1). Unfortunately, the dog developed right-sided congestive heart failure secondary to progressive pulmonary hypertension, which is currently under medical management with furosemide, pimobendan, and spironolactone.

## Discussion

Rivaroxaban, an oral human-specific factor Xa inhibitor, is used in dogs with a hypercoagulable state from an array of underlying etiologies (1, 11, 12). Based on current studies and clinical expertise, rivaroxaban appears to be safe and effective in dogs, with minimal adverse effects reported (1, 14, 16). However, additional research, controlled trials, and/or pharmacovigilance reporting are needed due to the limited number of studies and sample size limitations that exist in the current veterinary literature. The reported adverse effects in dogs include minor bleeding and vomiting (14). Despite numerous reports of hepatotoxicity in humans after the administration of rivaroxaban and other factor Xa inhibitors, similar findings have not yet been reported in veterinary medicine (6–8, 17–19). It is important to note that although reports exist in the human medical literature, factor Xa-induced hepatoxicity is a relatively rare event.

The exact mechanism of injury secondary to rivaroxaban therapy in humans is currently unknown, although direct toxicity, idiosyncratic, and immunologic mechanisms have been proposed (6, 9, 20). In humans with acute rivaroxaban hepatotoxicity, hepatocellular hepatopathies are most frequently noted, although cholestatic and mixed hepatopathies are also reported (6, 7, 21). In one study, up to 55% of factor Xa-induced hepatoxicity cases were characterized as hepatocellular (7). The dog in this report developed a severe, primarily hepatocellular hepatopathy with cytologic findings consistent with toxic injury. In human studies, elevation of serum total bilirubin is also frequently noted in rivaroxaban-induced hepatotoxicity cases (7, 21, 22). Although mild, the total bilirubin from the reported dog became mildly elevated along with the hepatopathy, which improved after the discontinuation of rivaroxaban.

The dog in this report developed a hepatopathy that was first noted on labwork 15 days after beginning rivaroxaban. The clinical signs of anorexia and lethargy began approximately 6 days prior (Day 9). Licata et al. (7) reported the time between the initiation of treatment and the onset of hepatic injury to range from 2 to 180 days with a median of approximately 15 days, which is consistent with our case. The most specific indicator of a hepatopathy being caused by rivaroxaban administration is improvement after the discontinuation of the drug. (6, 23, 24). While prospective data on the time to resolution of labwork derangements is limited, multiple case reports note significant improvement in liver enzymes 2–4 weeks after rivaroxaban was discontinued (6, 22–25). In the reported dog, the hepatopathy completely resolved 8 weeks after rivaroxaban was discontinued; however, significant improvement was documented at 4 weeks. The possibility of a pre-existing subclinical hepatopathy altering the metabolism and elimination process of this drug resulting in an increased propensity for hepatotoxicity, cannot be entirely excluded.

While all factor Xa inhibitors have been implicated in hepatotoxicity cases, rivaroxaban may be the most common, according to recent studies (6, 18, 19, 21, 22, 26). One case report noted improvement of severe hepatopathy after the discontinuation of rivaroxaban and starting apixaban, a similar factor Xa inhibitor (22). Another report showed similar findings when switching the patient from rivaroxaban to tinzaparin (27). Due to the lack of overall data regarding factor Xa inhibitor use in veterinary medicine, it is uncertain whether patients experiencing rivaroxaban-induced hepatotoxicity would be able to tolerate another drug from this class. Switching affected dogs to a different factor Xa inhibitor may be a valid alternative treatment option. In this case, after discussion with the client, it was elected not to pursue alternative antithrombic therapy given that there was no diagnostic evidence that the dog was currently in a hypercoagulable state, and the owner was reluctant due to concern for the development of any potential adverse side effects.

While hepatotoxicity secondary to rivaroxaban was prioritized as the most likely differential for this dog, other etiologies cannot be completely excluded. This dog was placed on antibiotics during her hospitalization, which could have contributed to an improvement in liver enzymes in the event that the elevations were secondary to bacterial hepatitis or cholangiohepatitis. It is worth noting that the dog’s liver enzymes had begun to improve prior to starting any therapies, including antibiotics, and no evidence of infection was noted on cytology or culture. The dog continued to improve at home despite her owner discontinuing all medications, which may further support the presumed diagnosis of rivaroxaban-induced hepatotoxicity. While no additional toxin exposure was observed or reported, it cannot be completely ruled out. Ideally, additional diagnostics including a liver biopsy, would have been performed to further support this diagnosis. Unfortunately, this was not perceived to be clinically indicated due to patient improvement and was also not financially feasible for the owner.

Although not the primary focus of this case report, it is clinically important to acknowledge that polypharmacy can increase the risk of drug–drug interactions (28) and that maropitant may be overprescribed in veterinary medicine for dogs lacking overt gastrointestinal signs (29). The utility and safety of each therapeutic and pharmacologic intervention should be carefully considered.

## Conclusion

To the author’s knowledge, this is the first case report of suspected rivaroxaban-induced hepatotoxicity in a dog. This case provides precedence to advocate for improved and closer monitoring of dogs receiving factor Xa inhibitors. Dogs on rivaroxaban should be monitored for clinical signs and labwork changes consistent with hepatotoxicity. In cases of suspected hepatotoxicity with no other identifiable cause, a risk–benefit analysis should be performed, and discontinuation of rivaroxaban administration or alternative antithrombotic medications should be considered.

## Data availability statement

The original contributions presented in the study are included in the article/supplementary material, further inquiries can be directed to the corresponding author.

## Ethics statement

Written informed consent was obtained from the owner of the dog for the publication of this case report.

## Author contributions

AM: Conceptualization, Data curation, Formal analysis, Investigation, Methodology, Project administration, Resources, Software, Validation, Visualization, Writing – original draft, Writing – review & editing. CW: Conceptualization, Data curation, Formal analysis, Investigation, Methodology, Project administration, Resources, Software, Supervision, Validation, Visualization, Writing – original draft, Writing – review & editing. KP: Conceptualization, Data curation, Formal analysis, Investigation, Methodology, Project administration, Resources, Software, Supervision, Validation, Visualization, Writing – original draft, Writing – review & editing. AB: Conceptualization, Data curation, Formal analysis, Investigation, Methodology, Project administration, Resources, Software, Supervision, Validation, Visualization, Writing – original draft, Writing – review & editing. KH: Conceptualization, Data curation, Formal analysis, Investigation, Methodology, Project administration, Resources, Software, Supervision, Validation, Visualization, Writing – original draft, Writing – review & editing. KVP: Conceptualization, Data curation, Formal analysis, Investigation, Methodology, Project administration, Resources, Software, Supervision, Validation, Visualization, Writing – original draft, Writing – review & editing.
